# Assessing morbidity management and disability prevention for lymphatic filariasis in India

**DOI:** 10.5588/pha.26.0047

**Published:** 2026-08-24

**Authors:** M.A. Bashar, A. Singh, K.C. Prajitha

**Affiliations:** 1 All India Institute of Medical Sciences, Gorakhpur, Uttar Pradesh, India;; 2 National Vector Borne Disease Control Programme, Gorakhpur, Uttar Pradesh, India;; 3 International Union against Tuberculosis and Lung Disease (The Union), Paris, France.

**Keywords:** elimination, filariasis, lymphoedema, operational research, SORT IT

## Abstract

**BACKGROUND:**

Evidence on the implementation of morbidity management and disability prevention (MMDP) remains limited in India.

**OBJECTIVES:**

To assess characteristics of persons with lymphatic filariasis (PwLF) registered for MMDP services from 2023 to 2025 and factors associated with regular receipt of services, the quality of MMDP services at designated health facilities, and barriers and facilitators to MMDP services in Gorakhpur district.

**METHODOLOGY:**

An explanatory mixed-methods study. The quantitative component involved cohort review of routine programme data from 16 public health facilities and cross-sectional quality assessment of 10 health facilities using WHO Direct Inspection Protocol. The qualitative component involved interviews and focus group discussions with beneficiaries, programme staff, and frontline workers.

**RESULTS:**

Out of the 946 registered PwLF, 811 were eligible to receive annual MMDP services, of which only 172 (21.4%) regularly received it. Patients with stage III or higher lymphoedema were twice as likely to regularly receive the annual services. All facilities assessed provided either suboptimal or poor-quality services, with gaps in availability of guidelines, supplies, and staff knowledge. Key barriers included supply interruptions, stigma, travel costs, and staff workload while community health workers facilitated follow-up.

**CONCLUSION:**

MMDP services continuity and quality remain suboptimal, requiring strengthened supply chains, provider capacity, and community-based follow-up.

## Graphical abstract

**Figure:**
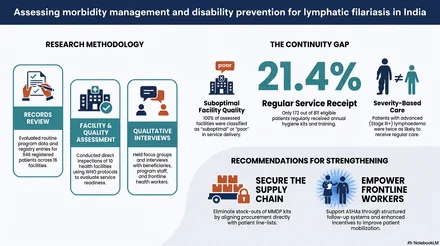

Lymphatic filariasis (LF) remains a leading cause of long-term disability among adults in many low- and middle-income countries. LF, caused by filarial parasites and transmitted through mosquitoes, leads to chronic lymphatic dysfunction manifesting as lymphoedema, elephantiasis, or hydrocele.^1^ The disease is closely linked to poverty, poor sanitation, and inadequate vector control. In 2023, approximately 657 million people across 39 endemic countries mainly in Africa and the WHO South-East Asia regions remained at risk of infection and required preventive chemotherapy.^1^ India bears one of the largest LF burdens globally: in 2023, there were an estimated 619,000 lymphoedema cases and 126,000 hydrocele cases across 348 endemic districts.^2,3^ Recognising the public health significance, India aligned itself with the WHO Global Programme to Eliminate Lymphatic Filariasis (GPELF), launched in 2000.^4^ GPELF rests on two pillars: interrupting transmission through mass drug administration (MDA) and alleviating suffering through morbidity management and disability prevention (MMDP) services. India aims to achieve elimination of LF by 2027, 3 years ahead of the global target.^1,4^

In 2004, India launched the elimination of lymphatic filariasis (ELF) programme, aligned with WHO’s GPELF. The programme was built on two pillars: MDA and MMDP. The MMDP strategy aims to ensure that all individuals with lymphoedema or hydrocele have access to appropriate care. This includes self-care training, management of acute episodes, hygiene promotion, and surgical intervention for hydrocele. Under the programme, mass campaigns for MDA were also initiated and MMDP services were provided to LF patients through Filaria units in public health facilities.^5^ However, while MDA coverage has improved over the years, MMDP – which is critical for reducing disability, improving quality of life, and meeting validation criteria for elimination – has received less operational attention.^6^ Evidence from endemic settings across the region suggests gaps in service availability, quality, provider capacity, and patient access.^7^ In India, these challenges are amplified in high-burden states such as Uttar Pradesh, Bihar, Odisha, and Jharkhand.^6,8^

Gorakhpur district, located in eastern Uttar Pradesh, is one of the 348 districts in India which are endemic for LF^2^ and is a high-burden setting. It has a large population of lymphoedema-affected individuals and a long epidemiological history of LF, including participation in early MDA pilot initiatives.^9,10^ Despite ongoing programmatic efforts, there is limited evidence on continuity and quality of care, and implementation challenges for delivering MMDP services at the designated health facilities in the region.^11^ Understanding these dimensions is crucial for strengthening service delivery and accelerating India’s pathway to LF elimination and requires a combination of quantitative and qualitative research methods.

With this background, this mixed-methods study was conducted to assess 1) characteristics of persons with lymphatic filariasis (PwLF) registered for receiving MMDP kits and training from 2023 to 2025 and factors associated with regular receipt of services; 2) quality of MMDP services at the designated health facilities; and 3) barriers and facilitators influencing implementation from the perspective of providers and beneficiaries.

## METHODS

An explanatory sequential mixed-methods design^12^ was used, comprising a quantitative component followed by a qualitative inquiry. The quantitative component included a non-concurrent cohort analysis of routinely collected programme data and a cross-sectional health-facility assessment to evaluate the quality of MMDP services. The qualitative part was a descriptive study using in-depth interviews (IDIs), key informant interviews (KIIs), and focus group discussions (FGDs). The study was conducted from January 2023 to December 2025 with data collection and analysis undertaken during 2025–2026.

### General setting

The study was coordinated by the Department of Community & Family Medicine, AIIMS Gorakhpur, in collaboration with the District Vector Borne Disease Control Programme, Gorakhpur. Uttar Pradesh, India’s most populous state, accounts for 17% of the national population and includes several LF-endemic districts.^13,14^ Gorakhpur district, located in eastern Uttar Pradesh, has a population of approximately 4.4 million across 19 community blocks and one municipal corporation.^13^ It is one of 348 districts endemic for LF and has a long history of endemicity. In 2023, the District Filaria Office of Gorakhpur documented nearly 2000 PwLF.^14^

### Specific setting

MMDP services in Gorakhpur are delivered through 32 designated health facilities – 13 urban primary health centres (PHCs) and 19 rural PHCs or community health centres. Each facility hosts an outpatient clinic staffed by a medical officer, supported by nurses and auxiliary nurse midwives (ANMs). Individuals with ailments including LF receive care through the outpatient clinics. Accredited Social Health Activists (ASHAs) mobilise newly detected PwLF for registration and follow-up care at these designated facilities.^15^

### Operationalisation of MMDP services

MMDP services follow three WHO-recommended components: i) access to a basic package of care including self-care training, MMDP kits, hygiene promotion, and referral for hydrocele surgery; ii) health-system strengthening for ongoing care; and iii) patient tracking and surveillance. ASHAs and ANMs conduct community surveys at regular intervals to identify and line-list PwLF, who are subsequently registered at designated facilities. Facilities are expected to provide MMDP hygiene kits (soap, towel, tub, bucket, mug, antifungal and antibiotic creams, nail-clippers, gauze, cold compress) and training on limb hygiene, acute attack management, and self-care practices annually.

## STUDY POPULATION AND SAMPLING

### Quantitative study

For assessment of regular receipt of MMDP services, all PwLF newly registered for receiving MMDP services at 16 (3 urban and 13 rural) out of the 32 designated health facilities between January 2023 and December 2025 were included. The remaining health facilities (n = 16) were excluded due to absence of ELF registers (n = 10) or follow-up data (n = 6). The quantitative cohort therefore represents all eligible records available from facilities with complete programme documentation. For quality assessment of MMDP services, a facility-level assessment was conducted using the WHO direct inspection protocol (DIP).^16^ A stratified random sampling technique was used to select facilities from the 32 designated facilities. Facilities were first stratified according to geographical location (urban/rural) and LF patient load (high/low burden) based on programme records, resulting in four strata: urban-high burden, urban-low burden, rural-high burden, and rural-low burden facilities. Two facilities were selected randomly from each stratum using a computer-generated random sequence. To ensure adequate representation of larger service delivery settings, two additional facilities were selected from strata with a higher number of PwLF registrations resulting in a total of 10 facilities (5 urban and 5 rural).

### Qualitative study

A total of 10 PwLF beneficiaries who received MMDP services were chosen for IDIs, and six programme officers (three programme managers and three medical officers) and two frontline workers (filaria inspectors) were chosen for KIIs. Beneficiaries participating in IDIs were selected through maximum variation sampling to reflect differences in gender, age, disease severity, adherence to follow-up, and rural–urban residence. Programme officers and frontline workers were selected through convenience sampling. Five FGDs were conducted: two with ASHAs (one each from urban and rural facilities), two with ANMs (one each from urban and rural facilities), and one with health supervisors selected from rural high- and low-burden facilities. Inclusion criteria for qualitative participants were willingness to participate and ability to provide informed consent; individuals unable to participate because of severe illness or unwillingness to consent were excluded.

### Data collection, variables and data source

Data were extracted from ELF registers available at the designated health facilities. Variables included demographic details, clinical characteristics (disease type and site, lymphoedema stage, duration of illness), treatment history (dates of registration, kit provision, training), and follow-up status. Inclusion for the cohort analysis required a unique registry entry with sufficient identifiers to assess follow-up; duplicate records and records without key identifiers were excluded. Facility assessments documented availability of trained staff, presence of guidelines and educational materials, water infrastructure, availability of medicines and supplies, patient-tracking systems, and staff knowledge on lymphoedema management. Facilities were scored across 14 indicators representing 6 quality domains. Each indicator was scored as ‘1’ if the component was present and ‘0’ if absent, based on direct observation and staff responses during the assessment, using the WHO DIP.^16^ The scoring system was selected because it provides a simple, transparent, and programmatically relevant measure of minimum service readiness.

Data were captured using Epicollect5^17^ and analysed using Stata 16.0 (Stata Corp LP, College Station, TX). Descriptive statistics summarised demographic and clinical characteristics. Regular receipt of MMDP services was operationally defined as receipt of all eligible annual follow-up MMDP kits and training following initial registration during the study period (2023–2025). This included two follow-ups for individuals registered in 2023 and one follow-up for individuals registered in 2024. Demographic and clinical characteristics associated with regular receipt of MMDP services were assessed using multivariable regression (modified Poisson with robust variance) and expressed using risk ratios and 95% confidence intervals. Facility performance scores, assessed using the WHO DIP questionnaire,^16^ were calculated by dividing the number of quality indicators met by the total 14 indicators. Facilities were classified as good (>75%), suboptimal (50%–75%), and poor (<50%).^16^

### Qualitative study

The quantitative findings informed the qualitative component of the study. All interviews and FGDs were conducted in Hindi, the local language, using a semi-structured interview guide by the principal investigator (male, medical doctor trained in qualitative research). Semi-structured interview guides were developed based on the study objectives and key domains identified from the quantitative findings. The guides included questions on access to MMDP services, follow-up practices and continuity of care, perceived benefits, and barriers to service utilisation at both individual and health-system levels. The interviews/discussion sessions were audio-recorded after obtaining written informed consent. For participants who did not consent to audio-recording, detailed field notes were taken during each interview. Efforts were made to capture verbatim expressions wherever possible. Interviews lasted 20–30 min, and FGDs lasted 40–50 min. Data collection continued until thematic saturation was achieved.

Electronic transcripts were prepared and translated to English on the same day of the interviews/discussions. Data were analysed using an inductive thematic analysis approach.^18,19^ Initial codes were generated inductively and organised into categories and overarching themes. Coding and theme development were conducted by the principal investigator (MAB), with input from KCP, an experienced qualitative researcher in the team, to minimise subjective bias. The analysis included independent reading of transcripts, iterative refinement of the codebook, comparison of codes across interviews, and resolution of discrepancies through discussion. Reflexive notes were maintained throughout the analytical process. For interviews not recorded, emphasis was placed on careful and consistent coding of detailed field notes. Findings are reported in accordance with the Consolidated Criteria for Reporting Qualitative Research (COREQ) guidelines.^20^

### Ethical statement

The study received ethics approval by Institutional Ethics Committee of AIIMS, Gorakhpur (IHEC/BMR/595 dated 10 November 2025). Written informed consent was obtained from all participants. Permission to access programme data and visit health facilities for assessing quality was granted by the Chief Medical officer, Gorakhpur district, Uttar Pradesh, India.

## RESULTS

A total of 1,189 records of PwLF were retrieved from 16 eligible public health facilities providing MMDP services in Gorakhpur district between 2023 and 2025. After excluding duplicate records, 946 PwLF were registered for receiving MMDP services during the study period and were included for analysis: 127 (13.4%) were registered in 2023, 693 (73.3%) in 2024, and 126 (13.3%) in 2025 (Figure 1).

**FIGURE 1.**
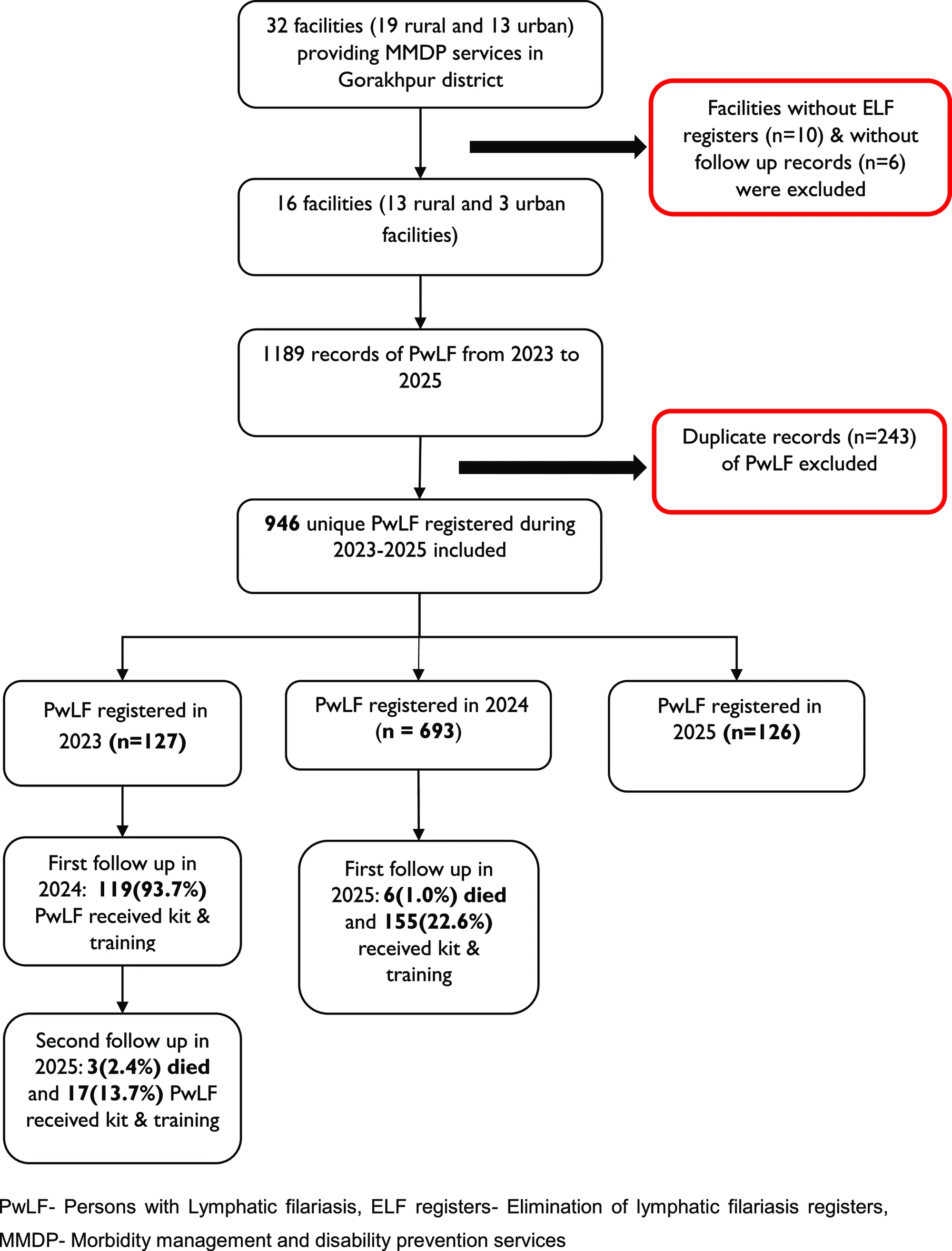
Flow diagram showing selection of records of PwLF for cohort analysis of regular receipt of annual MMDP services in Gorakhpur district, India, 2023–2025.

The mean (SD) age of participants was 55 (13) years, and most were female (643; 68%). The median duration of illness was 10 years (interquartile range: 7–20 years). Nearly all participants had lymphoedema involving the lower limbs (905; 95%). Over half were in advanced stages of disease, with 482 (53%) classified as stage III or higher. Detailed socio-demographic and clinical characteristics are presented in Table 1.

**TABLE 1.** Socio-demographic and disease-related characteristics of patients with lymphatic filariasis registered for morbidity management and disability prevention services in Gorakhpur district, 2023–2025.

Characteristics	Total	Year 2023	Year 2024	Year 2025
	N (%)	n (%) **^A^**	n (%) **^A^**	n (%) **^A^**
Number of patients registered and received first kit	946 (100.0)	127 (13.4)	693 (73.3)	126 (13.3)
Age in years				
Less than 30	58 (6.1)	5 (3.9)	47 (6.8)	6 (4.8)
30–59	466 (49.3)	59 (46.5)	345 (49.8)	62 (49.2)
60 and above	422 (44.6)	63 (49.6)	301 (43.4)	58 (46.0)
Sex				
Male	303 (32.0)	37 (29.1)	228 (46.3)	38 (30.2)
Female	643 (68.0)	90 (70.9)	465 (53.7)	88 (69.8)
Site of lymphoedema				
Right leg	444 (46.9)	56 (44.1)	322 (46.4)	66 (52.3)
Left leg	353 (37.3)	42 (33.1)	261 (37.7)	50 (39.7)
Both legs	108 (11.4)	25 (19.7)	77 (11.1)	06 (4.8)
Right arm	16 (1.7)	01 (0.8)	15 (2.2)	16 (16.9)
Left arm	07 (0.7)	01 (0.8)	05 (0.7)	01 (0.8)
Both arms	01 (0.1)	00 (0.0)	01 (0.1)	00 (0.0)
Right breast	05 (0.5)	01 (0.8)	04 (0.6)	00 (0.0)
Left breast	02 (0.2)	00 (0.0)	01 (0.1)	00 (0.0)
Both breasts	02 (0.2)	01 (0.8)	01 (0.1)	00 (0.0)
Others **^B^**	08 (0.6)	00 (0.0)	05 (0.7)	03 (2.4)
Stage of lymphoedema **^C^**				
Stage I	56 (6.1)	03 (2.4.)	53 (89.1)	00 (0.0)
Stage II	362 (39.7)	62 (48.8)	256 (72.8)	44 (34.9)
Stage III	421 (46.2)	47 (37.0)	295 (71.1)	79 (17.8)
Stage IV	44 (4.8)	09 (7.1)	33 (73.3)	02 (1.6)
Stage V	28 (3.1)	05 (4.0)	23 (88.9)	00 (0.0)
Stage VI	01 (0.2)	00 (0.0)	01 (0.0)	00 (0.0)
Stage VII	00 (0.0)	00 (0.0)	00 (0.0)	00 (0.0)
Not recorded	34 (3.6)	01 (0.8)	32 (4.6)	01 (0.8)
Setting				
Rural	910 (96.2)	127 (100.0)	658 (94.9)	125 (99.2.)
Urban	36 (3.8)	00 (0.0)	35 (5.1)	01 (0.8)
Patient’s follow-up status				
Continuing	937 (99.0)	124 (97.6)	687 (99.1)	26 (100.0)
Died	09 (1.0)	03 (2.4)	06 (0.9)	00 (0.0)

A Column percentages.

B Other sites as recorded in ELF registers.

C Staging is applicable only for lymphatic filariasis affecting limbs.

### Regular receipt of annual MMDP services

Among the 946 PwLF, 9 (1.0%) individuals died during the follow-up period and 126 (12.3%) were registered in 2025 and hence were not eligible to receive the annual follow-up MMDP services. Finally, 811 PwLF were eligible for receiving the annual follow-up MMDP services during the study period. Among these, only 172 individuals (21.2%) regularly received the annual MMDP hygiene kit and self-care training as per programme guidelines. In multivariable analysis, PwLF with stage III or higher lymphoedema were twice as likely (adjusted relative risk: 2.1 [1.5–2.8]) to receive the annual MMDP services regularly compared with those in earlier stages (Table 2).

**TABLE 2.** Factors associated with regular receipt of morbidity management and disability prevention services among persons with lymphatic filariasis registered between January 2023 and December 2024 in Gorakhpur district (n = 811).

Characteristics	N **^A^**	Regular receipt of MMDP services **^B^**	Unadjusted RR	95% CI	Adjusted RR	95% CI
		n (%) **^C^**				
Total	811	172 (21.2)	–	–	–	–
Age group (years)						
<30	52	6 (11.5)	1 (ref)	–	1 (ref)	–
30–59	404	88 (21.8)	1.9	(0.9–4.1)	1.06	0.8–1.4
≥60	355	78 (22.0)	1.9	(0.9–4)	1.52	0.7–3.3
Sex						
Male	258	49 (19.0)	1 (ref)	–	1 (ref)	–
Female	553	123 (22.2)	1.2	0.9–1.6	1.1	0.8–1.5
Site of lymphoedema						
Right leg	371	69 (18.6)	1 (ref)	–	1 (ref)	–
Left leg	302	71 (23.5)	1.3	0.9–1.7	1.3	0.9–1.7
Both legs	101	26 (25.7)	1.4	0.9–2.1	1.3	0.9–1.9
Others	34	4 (11.8)	0.6	0.3–1.7	0.9	0.4–2.4
Stage of lymphoedema^D^						
Stage II	56	1 (1.8)	1 (ref)	–	1 (ref)	–
Stage I	316	46 (14.6)	0.1	0.02–0.9	0.1	0.02–0.9
Stage 3–6^E^	408	120 (29.4)	**2.0**	**1.5–2.7**	**2.1**	**1.5–2.8**
Facility setting						
Urban	33	7 (21.2)	1 (ref)	–	1 (ref)	–
Rural	778	165 (21.2)	1.1	0.5–2.1	0.6	0.2–2

Boldness indicates that the values are statistically significant. MMDP = morbidity management and disability prevention; RR = risk ratio; CI = confidence interval.

A Of 820 individuals registered in 2023 and 2024, 9 individuals who died during the follow-up were excluded.

B Regular receipt of MMDP services: Received one MMDP hygiene kit (a bucket, mug, towel, tub, soap, nail-clippers, and antiseptic/antifungal creams) and self-care training in each calendar year after initial registration.

C Row percentages.

D Staging applicable for lymphoedema affecting limbs.

E Stage 3–6.

### Quality of MMDP services at health facilities

All 10 health facilities assessed were classified as providing either suboptimal (n = 8) or poor-quality (n = 2) MMDP services; none met criteria for optimal quality. Similar patterns were observed across urban and rural facilities. While several facilities had trained staff and essential medicines such as antiseptics and antibiotics, several critical components of lymphoedema management were absent across all assessed facilities (Table 3). Key gaps identified across facilities included absence of standard treatment guidelines, non-availability of essential lymphoedema care supplies, and poor staff knowledge related to recognition and treatment of acute attacks.

**TABLE 3.** Domain-wise tracer indicators and facility scores for quality of morbidity management and disability prevention services at 10 designated facilities in district Gorakhpur.^A^

Domain	Tracer indicator **^B^**	Urban facilities score (N = 5)		Rural facilities score (N = 5)	
		n	% **^C^**	n	% **^C^**
Trained staff	Staff trained in lymphoedema management	4	80	5	100
Case management materials	Guideline available	0	0	0	0
	IEC material available	2	40	3	60
Water infrastructure	Improved water source	4	80	4	80
Medicines and commodities	Antiseptics	5	100	5	100
	Antifungals	3	60	4	80
	Antibiotics	4	80	5	100
	Analgesics	5	100	5	100
	≥2 lymphoedema supplies	0	0	0	0
Patient-tracking system	Tracking system present	4	80	5	100
Staff knowledge	Identify lymphoedema symptoms	4	80	5	100
	Identify management strategies	3	60	3	60
	Identify acute attack symptoms	0	0	0	0
	Identify acute attack treatment	0	0	0	0
Total indicator score		38/70	54.3	44	62.8

A Using the WHO direct inspection protocol.

B Facility meeting all components specified by the indicator were assigned a score of ‘1’, otherwise ‘0’.

C Percentages were calculated as (n/N) × 100.

Qualitative analysis resulted in the emergence of three key themes (Figure 2).

**FIGURE 2.**
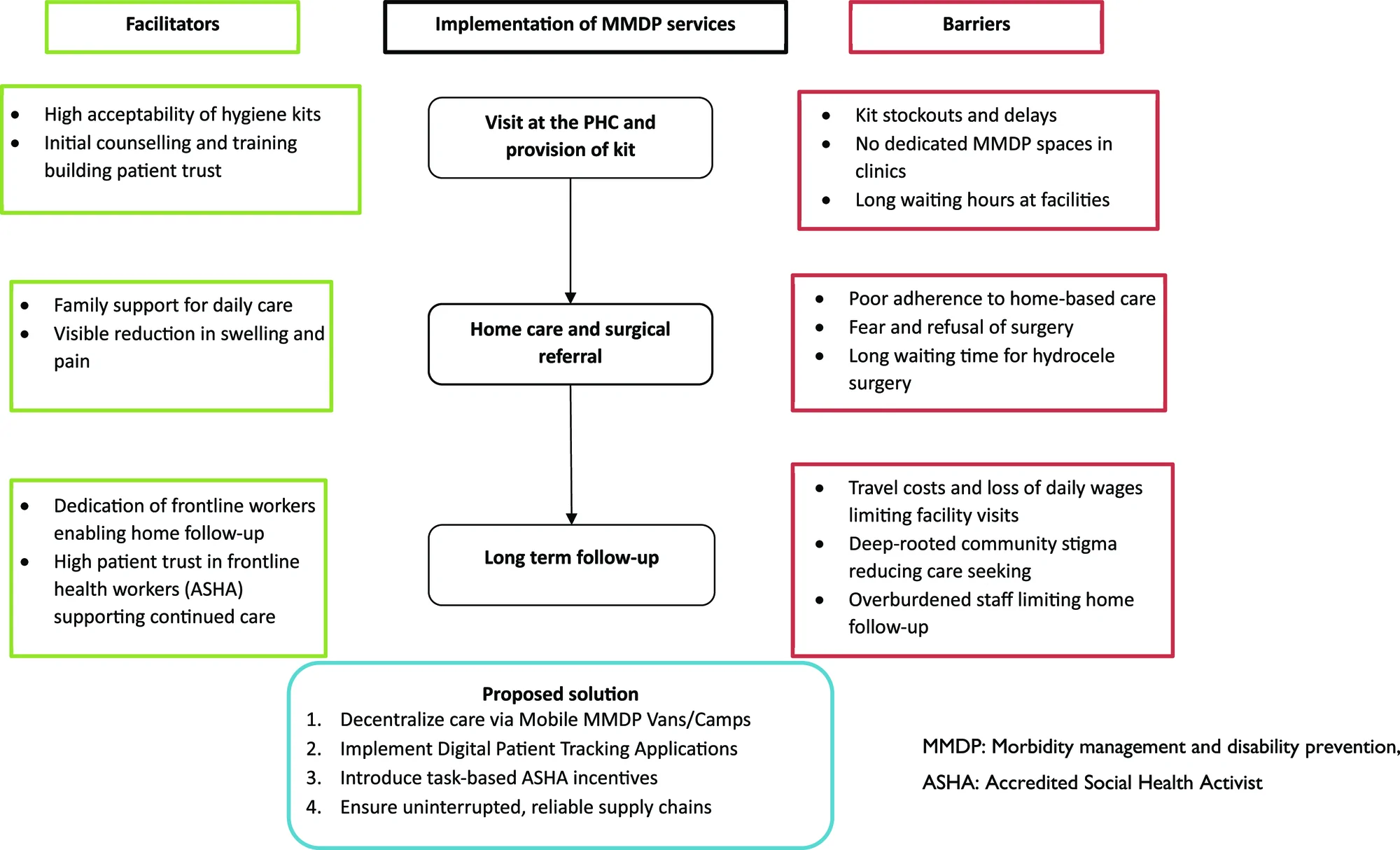
Facilitators, barriers, and proposed solutions for the implementation of morbidity management and disability prevention services for lymphatic filariasis in Gorakhpur. PHC = primary health centre.

#### Facilitators for MMDP services

Two key facilitators emerged: acceptability of MMDP components and support from community health workers. Beneficiaries reported noticeable improvements in daily functioning following receipt of the MMDP hygiene kit, training, and self-care guidance. Many described reductions in limb swelling and fewer acute attacks, which enabled them to resume routine activities. One beneficiary stated:

“My legs used to be so heavy that I couldn’t go to the farm. Now the swelling has reduced, and I don’t get acute attacks.”

Providers highlighted the pivotal role of community health workers in facilitating service utilisation. ASHAs were instrumental in identifying patients, mobilising them for care, and ensuring distribution of MMDP kits. As one ASHA explained:

“We go door-to-door, prepare lists, bring patients to camps, and distribute the MMDP kits.”

#### Implementation challenges

Implementation challenges were identified at both the health-system and patient/community levels. Health workers frequently reported irregular supply and stock-outs of MMDP kits, which disrupted continuity of services. One ASHA stated:

“Kit stock gets exhausted quickly, and new supplies arrive late.”

At the community level, stigma and social exclusion emerged as major barriers to care seeking. Beneficiaries described emotional distress resulting from discriminatory attitudes. A beneficiary shared:

My family is supportive, but in the community, they call me ‘hatti pair’ (elephant leg). It breaks my heart.

#### Strategies to strengthen implementation

Participants suggested several strategies focused on decentralised service delivery and health-system strengthening. Programme managers and medical officers emphasised the need for improved monitoring systems and stronger community-based implementation. Suggested interventions included digital line-listing, mobile MMDP camps, and enhanced incentives for ASHAs. A programme officer stated:

“Introduce digital line-listing, mobile camps, and increase ASHA incentives.”

These strategies were considered important to improve patient follow-up and continuity of care.

## DISCUSSION

This mixed-methods study provides important insights into the implementation of MMDP services for LF in an endemic district of northern India. The findings show that poor continuity, suboptimal facility readiness, and community-level barriers are interconnected rather than isolated problems, underscoring that while MMDP is a key component of LF elimination, its delivery within routine health systems remains suboptimal.

Only about one fifth of eligible PwLF in this study regularly received the annual MMDP kits and self-care training, indicating substantial gaps in continuity of care. This low coverage of follow-up MMDP services is concerning, given that LF is a chronic condition requiring sustained, long-term management to prevent disease progression and disability. The findings suggest that morbidity management remains weakly integrated into routine service delivery despite morbidity due to LF imposing a substantial social and economic burden, with greater programmatic emphasis continuing to be placed on transmission control through MDA.^21^ Similar findings have been reported from other endemic settings, reflecting a broader systemic challenge in sustaining chronic care within vertically oriented public health programmes and behavioural factors influencing care seeking and adherence.^22^

Disease severity was associated with regular receipt of MMDP services, with individuals having stage III or higher lymphoedema more likely to receive follow-up care. This may reflect increased care seeking by patients as disability advances and prioritisation of visibly severe cases by providers. While pragmatic in resource-constrained settings, this pattern raises equity concerns, as delayed engagement undermines the preventive intent of MMDP and may allow avoidable disease progression. Strengthening proactive follow-up and early engagement is therefore essential to move from a reactive to a preventive care model.

The health-facility assessments point to important deficiencies in the quality of MMDP service delivery. Gaps in availability of treatment guidelines, essential supplies, and provider knowledge suggest incomplete integration of morbidity management within primary health care systems. Similar implementation challenges have been reported in endemic settings, where limitations in training, supervision, and resource availability affect programme effectiveness.^23^ In addition, evidence from systematic reviews indicates that effective lymphoedema management depends on consistent access to hygiene-based care and appropriate provider support, highlighting the importance of maintaining minimum standards of care within health systems.^24^

This study has several strengths. First, it used a mixed-methods design, enabling a comprehensive assessment of regular receipt of MMDP services, quality of care, and implementation challenges. Second, the study utilised routinely collected programme data from multiple health facilities, reflecting real-world programme performance. Third, triangulation of quantitative and qualitative findings strengthened interpretation of implementation challenges. However, the reliance on routine programme records with incomplete documentation may have affected data completeness, and exclusion of half of the designated facilities because of missing registers and follow-up records likely introduced selection bias. The observational design also precludes causal inference. We have also adhered to Strengthening the Reporting of Observational Studies in Epidemiology (STROBE)^25^ and COREQ guidelines^20^ while reporting the study.

The study findings highlight several priorities for strengthening MMDP services. First, ensuring uninterrupted availability of MMDP kits and essential supplies is critical for improving continuity of care and sustaining patient engagement. Strengthening supply-chain mechanisms and aligning procurement with patient line-lists may help reduce recurring stock-outs. Second, gaps in provider knowledge and absence of standard guidelines indicate an urgent need for regular training, dissemination of simplified treatment protocols, and supportive supervision to improve quality of care. Third, weaknesses in record-keeping and patient tracking highlight the need to strengthen routine information systems, including exploration of digital line-listing and follow-up tools to support longitudinal care. Fourth, the qualitative findings underscore the central role of community health workers, particularly ASHAs, in facilitating patient identification, mobilisation, and follow-up. Strengthening their engagement through structured follow-up systems and appropriate incentives may enhance patient adherence and improve continuity of care. Fifth, barriers related to access to care, including travel costs, loss of daily wages, and long waiting times at facilities, highlight the need for more accessible and decentralised models of service delivery. Outreach-based services or community-level care approaches may help reduce these barriers and improve utilisation of MMDP services. Sixth, stigma and social exclusion associated with LF, as identified in the qualitative findings, continue to influence care-seeking behaviour and adherence to recommended practices. Addressing these social barriers through community awareness and engagement is essential for improving uptake of services.

## CONCLUSION

This study highlights important gaps in continuity and quality of MMDP services in a high-burden setting. Addressing these gaps through strengthened health systems and improved implementation strategies will be essential to improve patient outcomes and support the elimination of LF as a public health problem.
